# Challenging Clinical Therapeutic Approach to Urticarial Vasculitis: A Case Series

**DOI:** 10.3390/jcm14134580

**Published:** 2025-06-27

**Authors:** Fabio Artosi, Terenzio Cosio, Laura Diluvio, Gaetana Costanza, Filadelfo Coniglione, Maria Iacovantuono, Enrico Matteini, Elif Çağla Kaya, Sara Lambiase, Luca Bianchi, Elena Campione

**Affiliations:** 1 Dermatology Unit, Department of Systems Medicine, University of Rome Tor Vergata, 00133 Rome, Italy; costanza@med.uniroma2.it (G.C.); ematteini13@gmail.com (E.M.); elifcagla.kaya@gmail.com (E.Ç.K.); sara.lambiase@alice.it (S.L.); luca.bianchi@uniroma2.it (L.B.); elena.campione@uniroma2.it (E.C.); 2 Department of Laboratory Sciences and Haematological Sciences, Fondazione Policlinico Universitario “A. Gemelli” IRCCS, Università Cattolica Del Sacro Cuore, 00168 Rome, Italy; terenziocosio@gmail.com; 3 Unit of Dermatology, Tor Vergata Policlinic Foundation, University of Rome Tor Vergata, 00133 Rome, Italy; lauradiluvio@yahoo.it (L.D.); filadelfo.coniglione@uniroma2.it (F.C.); 4 Rheumatology, Allergology and Clinical Immunology, Department of Systems Medicine, University of Rome Tor Vergata, 00133 Rome, Italy

**Keywords:** urticarial vasculitis, leukocytoclastic, omalizumab, antihistamines, corticosteroid, treatment

## Abstract

**Background**: Urticarial vasculitis (UV) is a rare form of small-vessel vasculitis characterized by persistent urticarial lesions and systemic manifestations. It differs from chronic spontaneous urticaria (CSU) both clinically and pathogenetically, often requiring systemic therapy. Despite its complexity, no standardized treatment algorithm exists. **Methods**: We conducted a retrospective, monocentric, observational study on 11 patients diagnosed with UV at the Dermatology Unit of Tor Vergata University Hospital (Rome) between 2014 and 2024. Demographic, clinical, serological, and therapeutic data were collected from medical records. The therapeutic response was assessed using the Urticaria Control Test (UCT) score. **Results**: The cohort comprised predominantly women (91%), with a mean age at diagnosis of 52.8 years. Autoimmune thyroiditis was the most frequent comorbidity (64%). Hypocomplementemia was detected in only one patient (9%), who also had systemic lupus erythematosus. Antihistamines, while usually prescribed, showed limited efficacy since none of the patients achieved complete remission with monotherapy. Systemic corticosteroids demonstrated rapid and effective control in acute phases. Omalizumab produced variable responses, with two patients achieving a high response (HR) and one reaching complete remission (CR). Methotrexate and cyclosporine yielded inconsistent outcomes. Due to the heterogeneity and limited sample size, statistical analyses were not performed. **Conclusions**: UV presents with diverse clinical profiles and therapeutic responses. No treatment proved universally efficacious, but corticosteroids and omalizumab were effective in an acute and maintenance phase, respectively. Our findings underscore the importance of individualized treatment plans and the need for further studies to define predictive biomarkers and therapeutic strategies in UV.

## 1. Introduction

Urticarial vasculitis (UV) is a rare form of small-vessel leukocytoclastic vasculitis characterized by urticaria-like lesions that typically persist for more than 24 h, presenting with a burning sensation, itchiness, and pain [1,2,3]. Often, UV lesions resolve with residual hyperpigmentation [1,2]. Histopathology reveals perivascular neutrophilic infiltrates with nuclear debris, fibrinoid necrosis, and damage to post-capillary venules, distinguishing it from chronic spontaneous urticaria (CSU), which lacks vasculitic changes [1,2,3,4].

UV is considered a type III hypersensitivity reaction driven by the deposition of circulating immune complexes that activate the classical complement pathway [1,3]. Complement activation plays a pivotal role in its pathogenesis. Besides C3a andC5a—anaphylatoxins responsible for the recruitment and activation of neutrophils and mast cells—other components such as C1q, C4, and the membrane attack complex (C5b-9) have been implicated. Hypocomplementemia, particularly involving C1q, C3, and C4, is frequently observed in more severe cases and is associated with systemic manifestations [1,3].

Based on complement levels, UV is classified into normocomplementemic (NUV) and hypocomplementemic (HUV) subtypes [4]. While NUV often presents with isolated cutaneous symptoms and a more benign course, HUV is more frequently associated with systemic involvement, including arthralgia, arthritis, pulmonary disease, and features overlapping with systemic lupus erythematosus (SLE) [5,6]. Gastrointestinal symptoms, such abdominal pain, nausea, vomiting, and diarrhoea have been observed in up to one third of patients. Intestinal ischaemia is also a possible complication [6].

Although UV can be idiopathic, associations with autoimmune diseases (e.g., SLE, Sjögren’s syndrome), infections, hematologic malignancies, and certain drugs have been reported [1,7]. Vaccinations have also been implicated as potential triggers for the onset of UV, including recent reports following COVID-19 vaccination [8]. Given its protean manifestations and overlap with CSU, diagnosis requires a skin biopsy confirming leukocytoclastic vasculitis [3,9].

Treatment remains challenging due to the lack of standardized guidelines [1,3,10,11,12]. Second-generation antihistamines may provide symptomatic relief in mild NUV but are generally ineffective in controlling vasculitic inflammation [10,13]. Systemic corticosteroids are often required during flares or in patients with systemic involvement, but long-term use is limited by toxicity [3,14]. Immunosuppressants (e.g., azathioprine, methotrexate), biologics (e.g., rituximab), and other agents have been used off-label with variable success [1,3,7,15,16,17,18].

Omalizumab is a humanized monoclonal antibody directed against IgE, preventing its binding to the high-affinity receptor on basophils and mast cells. It is approved in Europe for allergic asthma, chronic rhinosinusitis with nasal polyps, and CSU [19]. Omalizumab has been favorably used in a limited number of NUV patients, granting some of the patients experienced brisk relapses after stopping the treatment [3,20].

To date, no validated clinimetric score specifically exists for assessing the severity and course of UV. However, instruments such as the Urticaria Control Test (UCT), originally developed to evaluate disease control and the quality of life in CSU, may also be applicable to patients with UV, as the symptoms assessed are common to both conditions. The UCT evaluates disease control over the preceding four weeks at each assessment point [21]. C-reactive protein (CRP) and the erythrocyte sedimentation rate (ESR), as markers of systemic inflammation, could help distinguish UV from CSU, when elevated at the baseline, but are not specific [22]. Laboratory tests most frequently requested by clinicians also include antinuclear antibody (ANA) and total IgE count to detect possible comorbidities such as SLE and to predict the response to omalizumab, although specific data for UV are lacking [23,24].

Herein, we describe a case series of 11 patients with UV managed at the University Hospital “Tor Vergata” in Rome between January 2014 and November 2024, aiming to provide insights into the clinical features, treatment approaches, and therapeutic outcomes in this heterogeneous disease.

## 2. Materials and Methods

### 2.1. Study Design

Based on the information collected from patients’ charts, and laboratory and biopsy records, we performed a retrospective, descriptive, monocentric study about the epidemiological, clinical, and therapeutic response of all the patients affected by UV that we visited at the Dermatology Unit of “Tor Vergata” University Hospital of Rome since 2014. All patients underwent a confirmatory skin biopsy on an active lesion. In cases where the pathologist did not issue a definitive diagnostic report, the presence of leukocytoclasia and/or fibrin deposits was considered histopathological confirmation of UV [1]. We obtained from our internal database the following data: demographic data, comorbidities, presence or absence of diseases or signs that are known to be associated with UV, the sequence of treatments undertaken in relation to each patient’s clinical response, and the baseline C3, C4, C1q, IgE, ANA, VES, and CRP blood levels. The study was conducted following the ICH/GCP guidelines and in compliance with the current regulations relating to studies on off-label drugs and was approved by the Institutional Review Board’s (IRB) Independent Ethical Committee Tor Vergata University Hospital (N.82.25 CET2 PTV approved on 13 March 2025). Written informed consent was obtained from the patients to publish this case series and any accompanying images.

### 2.2. Scores

The UCT questionnaire defines the criteria for assessing the clinical control of urticaria as follows: a score of 16 indicates complete disease control; an increase of 3 points or less reflects a minimal response; and an increase of at least 6 points indicates a marked response (Table S1) [20]. To better stratify therapeutic outcomes, we established a clinical criterion for therapeutic response based on the UCT. The response categories considered in this study are as follows:

- Worsening of disease: reduction in UCT value compared to the baseline visit.
- No response: no change in UCT value compared to the baseline visit.
- Low response: increase in UCT score for a form 1–3 compared with the baseline visit, with residual disease activity.
- Moderate response: increase in UCT score for a form 4–6 compared with the baseline visit, with residual disease activity.
- High response: increase in the UCT score for a form 7 or higher compared with the baseline visit, with residual disease activity.
- Complete response: a score of 16 at the UCT questionnaire.

### 2.3. Comorbidity Assessment

Gastrointestinal involvement was considered positive when one or more of the following symptoms—abdominal pain, nausea, vomiting, or diarrhea—occurred within one month before or after the diagnosis of UV [1]. Thyroid involvement was defined as the presence of autoimmune thyroid disease confirmed by both serological and ultrasound findings. Positivity for joint involvement was defined by the presence of arthralgia and/or arthritis raised in a period ranging from one month before to one month after the diagnosis of UV.

## 3. Results

### 3.1. Characteristics of UV Cohort Patients

We identified 11 patients, including 10 women and one man. Each patient exhibited different comorbidities alongside UV and underwent various therapeutic approaches. The patients were diagnosed with UV between 2014 and 2024, with a mean age at diagnosis of 52.8 years. With the exception of patient No. 7, all the patients had at least two comorbidities (Table 1), the most common being autoimmune thyroiditis, followed by asthma/allergic rhinitis, affecting 64% and 45% of patients, respectively. Documented *Helicobacter* (*H*.) *pylori* infection, peptic ulcer disease, hypertension, diabetes, gastroesophageal reflux disease, and hypercholesterolemia each affected 18% of patients, whereas other comorbidities were observed in 9% of patients. Patient No. 11 had a diagnosis of SLE and developed urticarial-like lesions clinically consistent with urticarial vasculitis UV, which were confirmed as leukocytoclastic vasculitis on histopathological examination.

Below are cases of patients in whom there was a temporal overlap (within 30 days) between the onset of UV and the following comorbidities:

- Patient No. 4, who developed UV following *H. pylori* infection, with remission observed after eradication therapy.
- Patient No. 6, who experienced the onset of vitiligo concomitantly with UV.
- Patient No. 8, who developed transient diplopia due to ocular myasthenia gravis at the onset of UV.

### 3.2. Laboratory Findings

Only Patient No. 11 (9%) had hypocomplementemic UV (Table 2); in all the other cases, the C3, C4, and C1q levels at diagnosis were within the normal ranges. The total IgE levels (Table 2) were elevated at the baseline in Patients No. 2, No. 3, and No. 7, corresponding to 27% of the cases. Notably, Patient No. 3 exceeded the cut-off by only 0.5 IU/mL. ANA positivity was observed in three patients (27%): No. 1, No. 6, and No. 11 (Table 1). However, only Patient No. 11 showed significantly elevated ANA titers (1:320), whereas the titers in Patients No. 1 and No. 6 were at the lower sensitivity threshold (1:80). Two patients (18%) had elevated CRP levels at the baseline (Patients No. 1 and No. 6), with Patient No. 6 also exhibiting elevated ESR.

### 3.3. Treatments

A summary of demographic characteristics, comorbidities, and therapeutic responses to each drug is provided in Table 1.

#### 3.3.1. Antihistamines

All the patients received at least one antihistamine therapy, either alone or in combination with other treatments. Seven patients (64%) received antihistamine monotherapy at some point during their treatment course, but none achieved complete remission. Only one patient (9%) treated exclusively with antihistamines achieved a high therapeutic response (HR); another exhibited a low response (LR), two (18%) had a moderate response (MR), and three (27%) showed no response (NR). One of these patients, after tripling the standard antihistamine dose, progressed from LR to MR. In one case (9%), a patient achieved a complete response (CR) with a combination of loratadine and H. pylori eradication therapy.

#### 3.3.2. Systemic Corticosteroids

Eight patients (73%) received at least one course of systemic corticosteroids (SCCS), including four as first-line therapy, either alone or in combination with other drugs. Two patients (No. 3 and No. 6, 18%) underwent multiple courses of corticosteroids lasting 4–6 months each, with responses ranging from LR to MR. Even when administered alongside azathioprine, as in Patient No. 5, only an LR was achieved. In Patient No. 6, an initial LR was observed with antihistamines, but a subsequent HR was achieved with omalizumab, particularly during intermittent SCCS use. Similarly, Patient No. 7 demonstrated an LR in combination with antihistamines. In Patients No. 8, No. 9, and No. 10, SCCS provided an excellent response that could not be attained with antihistamines alone. Specifically, Patients No. 8 and No. 10 reached CR and were able to discontinue systemic therapy after a slow SCCS taper. In Patient No. 9, despite achieving HR with SCCS, omalizumab was introduced to maintain HR while reducing corticosteroid toxicity.

In Patient No. 11, SCCS provided an excellent response (HR) as the first effective therapy after failure with antihistamines, hydroxychloroquine, and methotrexate. After five months, to minimize the potential side effects and achieve adequate control of concurrent SLE, anifrolumab, a monoclonal antibody approved in Europe for SLE that binds to the subunit 1 of the type I interferon receptor, was initiated [25]. Among the eight patients treated with SCCS, a rapid clinical response was observed within 24 h of treatment initiation, confirming corticosteroids as the most rapidly acting therapy in this cohort.

#### 3.3.3. Omalizumab

Omalizumab was administered to five patients (45%), as monotherapy in one case and as add-on therapy with antihistamines or SCCS in four cases. The clinical responses varied significantly among patients and over time. Omalizumab was never used as a first-line treatment. The clinical responses after 2–3 months of therapy were as follows:

- One MR in combination with antihistamines (Patient No. 2).
- One worsening (W) in combination with antihistamines (Patient No. 5).
- Two HRs, one with concomitant antihistamines (Patient No. 9) and one without (Patient No. 3).
- One LR when administered alone, but HR when combined with SCCS.

In Patient No. 2, an initial MR led to continued treatment for 17 months, ultimately achieving CR and discontinuation of therapy. In Patient No. 3, after achieving HR, therapy was discontinued, but a significant UV relapse with severe angioedema occurred within six months, necessitating SCCS therapy and subsequent reintroduction of omalizumab. This patient remained on omalizumab therapy with sustained HR for over 82 months. Patient No. 5 continued omalizumab therapy and, after initial worsening, exhibited fluctuating disease activity with responses ranging from LR to MR over 78 months. Patient No. 6 discontinued omalizumab after 66 months due to inadequate efficacy and mild progressive UV worsening. Patient No. 9 was on omalizumab plus antihistamines for over two months, maintaining HR comparable to previous SCCS and antihistamine therapy. Thus, two out of five patients continued omalizumab therapy with the efficacy ranging from LR to HR, one discontinued due to CR, and two discontinued due to primary or secondary inefficacy. The omalizumab dosage for all patients was 300 mg subcutaneously every four weeks.

#### 3.3.4. Methotrexate

Three patients (27%) received methotrexate (MTX) once weekly for UV, but none as first-line therapy. Patient No. 3 received MTX 15 mg weekly for over three months, though the exact duration was unclear as therapy occurred between 2016 and 2017, resulting in LR and subsequent discontinuation. Patient No. 6 responded excellently to MTX 15 mg, achieving HR, nearly CR. The MTX dose was tapered to 7.5 mg weekly without clinical worsening, and the patient remained on therapy 15 months after initiation. Patient No. 11 showed no response to MTX 10 mg weekly (NR) and discontinued therapy after three months.

#### 3.3.5. Hydroxychloroquine

Hydroxychloroquine (HCQ) was used in three patients (27%), always as monotherapy. The responses included LR in two cases and NR in another, with exposure ranging from one to six months. HCQ was used in patients with multiple therapy failures: Patient No. 3 responded only to omalizumab, Patient No. 5 exhibited a poor response to all attempted treatments, and Patient No. 11, with SLE, responded only to SCCS and anifrolumab.

#### 3.3.6. Cyclosporine

Cyclosporine was used in two patients (18%), never as a first-line treatment. MR was achieved after more than three months in Patient No. 3 and 10 months in Patient No. 5. In Patient No. 3, cyclosporine yielded better responses than previous corticosteroid therapy, though not fully satisfactorily. The patient developed hypertension, leading to treatment discontinuation. In Patient No. 5, despite only a moderate response, cyclosporine remained the most effective therapy within a three-month observation period.

#### 3.3.7. Other Drugs

Other treatments used in this study included azathioprine (Patient No. 5) and anifrolumab (Patient No. 11). Azathioprine yielded LR in a patient with multiple therapy resistances, while anifrolumab provided HR with concurrent suppression of both UV and SLE activity.

## 4. Discussion

Urticarial vasculitis is a rare and complex disease characterized by distinct histopathological features and associated comorbidities that vary from patient to patient [1]. Currently, no approved therapy exists for this condition, although several systemic drugs are commonly used in clinical practice [13]. In this retrospective study, we assessed different off-label therapeutic scenarios and observed that no single drug could be identified as the primary treatment for UV, as each patient exhibited a distinct response to the available therapies due to individual differences in comorbidities, lifestyle, and the small sample size. UV is an immune complex-mediated disease classified as a type III hypersensitivity reaction, in which antibodies form complexes with antigens (either autologous or exogenous in origin) and activate the classical complement pathway, leading to the generation of C3a and C5a fragments and subsequent mast cell degranulation. Mast cells release tumor necrosis factor-alpha, prostaglandins, histamine, heparin, platelet-activating factor, neutrophil chemotactic factor A, proteases, and tryptase [1,12,26]. In our retrospective study, only one female patient exhibited baseline C3, C4, and C1q levels below normal, and she was also diagnosed with SLE. All the other patients had normal values for the complement proteins under investigation. A total of 64% of the patients in this study had autoimmune thyroiditis, whose association with UV is broadly known [26]. Overall, 64% of our patients exhibited an autoimmune comorbidity, with conditions such as vitiligo, antral gastritis, ocular myasthenia gravis, generalized myasthenia gravis, SLE, and celiac disease. This finding is supported by the autoimmune context in which UV frequently develops, driven by antigen–antibody complex formation [26,27]. However, it is noteworthy that only three (27%) patients were ANA-positive, with just one patient exhibiting a high titer, whereas the other two had titers near the lower sensitivity threshold of the laboratory tests. Systemic inflammatory markers were elevated at the baseline in only two patients, both diagnosed with NUV, suggesting that their utility in distinguishing UV from CSU was less evident than previously reported [4].

We believe that the 4-week recall period of the UCT provides a sufficiently long timeframe to more accurately capture the disease course of urticarial vasculitis (UV), compared to other instruments such as the Urticarial Vasculitis Activity Score, which assesses the disease activity over approximately 24 h prior to the visit. Furthermore, the latter score has not yet been validated [28].

One of the key diagnostic clues differentiating UV from CSU is the poor or absent response to second-generation antihistamines, even at high doses [10]. None of our patients achieved complete remission with antihistamine monotherapy. Of the seven patients who received antihistamines at some point in their therapeutic course, only one exhibited a good response, whereas the others displayed nearly uncontrolled disease activity. In our study, antihistamines were frequently administered as add-on therapy alongside systemic treatments, largely following the treatment paradigm of CSU, with variable responses among patients, mainly influenced by the systemic therapy co-administered.

The vasculitic nature of urticarial vasculitis (UV) involves the participation of multiple immune components, including eosinophils, neutrophils, mast cells, basophils, lymphocytes, and immune complexes, which contribute to the damage of small blood vessel walls [26]. Consequently, the blockade of the histamine H1 receptor on mast cells may provide only limited symptomatic relief of pruritus in some patients. This contrasts with CSU, a condition primarily driven by mast cell activation, in which the cross-linking of the high-affinity IgE receptor (FcεRI) on cutaneous mast cells by IgE and IgG autoantibodies triggers intracellular signaling pathways that culminate in the release of vasoactive mediators, particularly histamine, the principal effector of urticarial symptoms [29]. The response to systemic corticosteroid therapy varied, with low to moderate effectiveness in four patients and high effectiveness in four others. In all cases in which SCCS were added to an antihistamine regimen, a marked clinical improvement was observed, as seen in Patients No. 6, 8, 10, and 11. Although antihistamines were administered to all the patients, either as monotherapy or in combination with other treatments, their overall efficacy appeared limited. Notably, none of the patients treated with antihistamines alone achieved complete remission, and high therapeutic responses were rare. Interestingly, in Patient No. 6, SCCS monotherapy initially produced an unsatisfactory response, whereas upon the introduction of omalizumab, the SCCS response became high. This suggests that omalizumab may have sensitized the disease to corticosteroid therapy. Omalizumab may enhance the immune system’s responsiveness to corticosteroids by reducing baseline inflammation, inhibiting the IgE–mast cell axis, and modulating the cytokine and cellular profile. The reduction in free IgE levels leads to the downregulation of FcεRI, thereby decreasing the activation potential of effector cells [30,31]. The proposed mechanism involves the activation of protein phosphatase 2A (PP2A), which counteracts eosinophil peroxidase-mediated inactivation, thereby improving the corticosteroid responsiveness [32].

In our study, SCCS produced a rapid and high response. Four patients achieved HR with SCCS, two of whom reached CR and were able to discontinue systemic therapy for UV. The other two high responders remained on anifrolumab and omalizumab to control concurrent SLE and UV, respectively, while reducing systemic corticosteroid toxicity.

Five patients were treated with omalizumab, with or without concomitant antihistamine therapy, with highly variable responses ranging from clinical worsening to two cases of HR, with partial improvement in another patient. Notably, in Patient No. 2, although no substantial improvement was observed in the early months of omalizumab treatment, after nearly 18 months of therapy, the drug was discontinued due to complete remission. It remains unclear whether omalizumab influenced the natural course of the disease, but it is important to consider that UV may exhibit a self-limiting nature over time. In Patient No. 3, omalizumab did not seem to alter the natural progression of UV. This patient was treated with omalizumab for three months with excellent disease control, followed by discontinuation. However, a steady worsening of UCT scores was observed, eventually reaching values worse than baseline. Due to severe angioedema, SCCS therapy was reintroduced, followed by a slow taper in conjunction with omalizumab reintroduction, leading to an excellent and sustained therapeutic response that has persisted for 82 months. A similar scenario was observed in Patient No. 6, who experienced slow, progressive worsening of UV while on omalizumab for over 66 months.

In the case of Patient No. 5, after initial clinical worsening with omalizumab, we opted to continue treatment in light of previous therapeutic failures and its favorable safety profile [33]. This patient remained on omalizumab therapy, exhibiting fluctuating disease control over 78 months, with response levels comparable to or slightly better than previous, more toxic therapies [34].

Among the five patients treated with omalizumab, those with normal serum IgE levels exhibited weak or absent responses in two out of three cases, whereas both patients with elevated baseline IgE levels achieved excellent responses. Although our study lacks the statistical power to definitively associate omalizumab efficacy in UV with elevated baseline IgE levels, our findings support previous observations [35]. Similar to CSU patients, some authors have reported that UV patients with high baseline IgE levels respond better to omalizumab [36]. By reducing free IgE levels, omalizumab may decrease the formation of IgE-containing immune complexes that deposit on vessel walls, a hallmark of vasculitic processes. This mechanism could be particularly relevant in NUV, where immune complex-mediated vascular damage is implicated [30].

Three patients were treated with MTX for UV. Two of them showed minimal clinical benefit despite receiving therapy for over three months. In contrast, the third patient experienced a rapid and marked improvement, achieving near-complete remission within days of the first injection. In this case, the MTX dose was successfully tapered from 15 mg/week to 7.5 mg/week without any loss of efficacy.

In comparison, HCQ yielded even less promising results. None of the three patients treated with HCQ achieved a satisfactory response. It is important to note, however, that HCQ was administered to patients with multiple prior treatment failures, which may have limited its apparent efficacy.

Cyclosporine also failed to produce substantial clinical improvement. Nevertheless, its rapid onset of action may offer utility as a short-term alternative to SCCS for managing wheal activity.

Interestingly, anifrolumab achieved complete symptom control in a young, treatment-refractory patient with NUV and SLE, suggesting a potential role for interferon pathway inhibition in selected cases.

This retrospective study is based on a cohort of only 11 patients, reflecting the rarity of urticarial vasculitis (UV). Due to the small sample size and the heterogeneity of treatment regimens administered over extended and variable time periods, conducting a conclusive statistical analysis on treatment efficacy is not feasible.

## 5. Study Limitations

Given the rarity of UV, the cohort included only 11 patients, all evaluated at a single center over a long time period. The heterogeneity in clinical presentations, as well as in the treatments administered and their variable responses, precluded the possibility to make a statistical analysis, underscoring the need for studies with larger patient populations.

In the absence of a validated clinimetric score specific to UV, we employed the UCT, a tool validated for CSU, which assesses disease control over the preceding four weeks [20]. This timeframe is more appropriate for capturing the fluctuating course of UV—including flares and remissions—compared to other instruments such as the Urticarial Vasculitis Activity Score, which evaluates symptoms over the previous 24 h and is not still validated [28].

## 6. Conclusions

There is no single optimal treatment for UV, nor is there a prototypical UV patient. Our study highlights the substantial variability in patient responses to the same drugs and, in some cases, to the same drug at different times in the same patient. Omalizumab could be a promising treatment, with no associated adverse events reported in our patients. Systemic corticosteroids were useful in our cohort due to their rapid action in acute or exacerbation phases of UV. However, there is a pressing need for better profiling of UV clinical subsets through studies with larger patient cohorts to more precisely determine the most effective treatment strategies.

## Figures and Tables

**Table 1:** Summary of demographic, clinical, and therapeutic characteristics of enrolled patients. AA = angioedema; As/R = asthma and/or allergic rhinitis; CR = complete remission; CyS = systemic cyclosporine; Gi = gastrointestinal involvement; HCQ = hydroxychloroquine; HR = high response; Jo = joint involvement; LR = low response; MR = moderate response; MTX = systemic methotrexate; NR = no response; R = relapse; SCCS = systemic corticosteroid; TrC = treatment continuation (the patient continues the treatment); TrD = treatment discontinuation (the patient discontinues the treatment); Th = thyroid involvement; W = worsening of disease; Wh = wheals; (“number”)→ = number of months elapsed until the occurrence of the next event; - = absence of the sign/symptom; + = presence of the sign/symptom.

PATIENTS	Date of Diagnosis	Comorbidities	AA	Wh	Gi	Th	As/R	Jo	Sequence of Treatments
No. 1, 52-year-old female	2023, June	Hypertension, celiac disease	-	+	+	+	-	-	Cetirizine (4)→ HR; TrC.
No. 2, 79-year-old female	2022, December	Peptic ulcer disease	+	+	+	+	-	-	Ebastine (1)→ LR→ Ebastine 3/die (1)→ MR→ Omalizumab + ebastine (3)→ MR→ TrC (17)→ CR→ TrD
No. 3, 60-year-old female	2015, April	Severe asthma, glucose-6-phosphate dehydrogenase deficiency, diabetes mellitus, hypertension, melanoma, chronic kidney disease, antral gastritis, gastroesophageal reflux disease, obstructive sleep apnea	+	+	-	+	+	-	SCCS→ LR, → CyS→ MR with SE; MTX 15 mg→ LR, HCQ→ LR; SCCS (4)→ MR; omalizumab (3)→ HR; TrD (6)→ W; SCCS (6)→ LR; omalizumab+rupatadine (82)→ HR; TrC
No. 4, 36-year-old, male	2023, February	Gastroesophageal reflux disease, newly diagnosed helicobacter pylori infection	+	+	+	+	+	+	Fexofenadine(2)→ MR→loratadine + eradication therapy for helicobacter pylori (2)→ CR; TrD
No. 5, 56-year-old, female	2014, August	Thyroid carcinoma, borderline ovarian cancer, serous chorioretinopathy	+	+	-	-	+	+	Rupatadine since 2015 as add-on therapy to: Azathioprine + SCCS (4)→ LR; HCQ(8)→ LR; CyS (10)→ MR; omalizumab (2)→ W; TrC (78)→ variable response, from LR to MR; TrC
No. 6, 75 year-old, female	2017, July	Hypercholesterolemia, vitiligo (arising at the same time as UV), dysphagia	+	+	+	+	-	+	SCCS + rupatadine (8)→ LR; rupatadine + omalizumab + periodic intake of SCCS (66)→ LR, HR when taking SCCS; MTX 15 mg (3)→ HR; MTX 12.5 mg (3)→ HR; MTX 10 mg (3)→ CR; MTX 7.5 mg (6)→ HR, TrC
No. 7, 30-year-old, female	2018, November		+	+	-	-	+	-	Cetirizine (3)→ NR; SCCS + hydroxyzine + fexofenadine (4)→ LR; rupatadine 2/die (4)→ MR; TrC.
No. 8, 66-year-old, female	2024, May	Diplopia (ocular myasthenia gravis) arising simultaneously with UV, diabetes mellitus	-	+	-	+	-	-	Cetirizine + bilastine (2)→ MR; rupatadine + SCCS with slow tapering (2)→ CR; TrD
No. 9, 62-year-old, female	2024, April	Hypercholesterolemia	+	+	-	+	-	-	Hydroxizine (1)→ NR; cetirizine (1)→ NR; SCCS + rupatadine (2)→ HR; omalizumab + rupatadine (2)→ HR, TrC
No. 10, 44-year-old, female	2024, July	Generalized myasthenia gravis	+	+	+	-	-	-	SCCS + rupatadine (2)→ HR, TrD (2)→ CR
No. 11, 21-year-old, female	2023, July	Immune trombocytopenic purpura, systemic lupus erythematosus	+	+	+	-	-	+	Chlorphenamine→ NR; HCQ(1)→ NR; MTX 10 mg (3)→ NR; SCCS (5)→ HR, TrC; Anifrolumab (5)→ HR, TrC

**Table 2:** Blood values of complement, total IgE, ANA, ESR, and CRP at baseline. Normal values for each blood parameter in brackets. ANA: antinuclear antibody; CRP = C-reactive protein ESR = erythrocyte sedimentation rate; IgE = immunoglobulin E.

PATIENTS	Baseline Complement Levels	Baseline IgE Levels	Baseline ANA Levels	Baseline ESR and CRP Levels
No. 1, 52-year-old female	C3: normal C4: normal C1q: normal	24 IU/mL (<100 IU/mL)	Positive, 1:80	CRP: 7 mg/L (0–5 mg/L) ESR: 42 (2–25 mm/h)
No. 2, 79-year-old female	C3: normal C4: normal C1q: normal	135 IU/mL (<100 IU/mL)	Negative	CRP: 5 mg/L (0–5 mg/L) ESR: 28 mm/h (0–35 mm/h)
No. 3, 60-year-old female	C3: normal C4: normal C1q: normal	88.5 IU/mL (<88 IU/mL)	Negative	CRP: 3.9 mg/L (0–5 mg/L) ESR: 21 (0–25 mm/h)
No. 4, 36-year-old male	C3: normal C4: normal C1q: normal	11.8 IU/mL (<100 IU/mL)	Negative	CRP: 3 mg/L (<5 mg/L) ESR: 14 mm/h (<25 mm/h)
No. 5, 56-year-old female,	C3: normal C4: normal C1q: normal	24 IU/mL (<100 IU/mL)	Negative	CRP: <1 mg/L (<5 mg/L) ESR: 12 mm/h (2–25 mm/h)
No. 6, female, 75-year-old,	C3: normal C4: normal C1q: normal	3.11 UI/mL (0–87 IU/mL)	Positive, 1:80	CRP: 9 mg/L (<5 mg/L) ESR: 2 mm/h (2–30 mm/h)
No. 7, 30-year-old female,	C3: normal C4: normal C1q: normal	448 IU/mL (<100 IU/mL)	Negative	CRP: 2.3 mg/L (<5 mg/L) ESR: 24 mm/h (<25 mm/h)
No. 8, 66-year-old, female	C3: normal C4: normal C1q: normal	14 UI/mL (0–87 UI/mL)	Negative	CRP: 7.7 mg/L (<5 mg/L) ESR: 14 mm/h (2–30 mm/h)
No. 9, 62-year-old, female	C3: normal C4: normal C1q: normal	22,10 UI/mL (0–87 UI/mL)	Negative	CRP: <0.5 mg/L (0–3 mg/L) ESR: 15 (0–15)
No. 10 24-year-old, female	C3: normal C4: normal C1q: normal	14 IU/mL (0–87 UI/mL)	negative	CRP: 1.6 mg/L (<5 mg/L) ESR: 11 mm/h (2–30 mm/h)
No. 11 21-year-old, female	C3: 30 mg/dL (83–193 mg/dL) C4: <2.9 mg/dL (15–57 mg/dL) C1q: 85 mg/dL (150–300 mg/dL)	39 IU/mL (0–87 UI/mL)	positive, 1:320	CRP: 3.5 mg/L (<5 mg/L) ESR: 14 mm/h (2–30 mm/h)

## Data Availability

The original contributions presented in this study are included in the article/Supplementary Material. Further inquiries can be directed to the corresponding author(s).

## Supplementary Materials

The following supporting information can be downloaded at: https://www.mdpi.com/article/10.3390/jcm14134580/s1, Table S1: URTICARIA/ANGIOEDEMA (Hives/Swelling) CONTROL TEST.

## Abbreviations

The following abbreviations are used in this manuscript:

AA	Angioedema
ANA	Antinuclear antibody
As/R	Asthma and/or allergic rhinitis
CR	Complete response
CRP	C-reactive protein
CSU	Chronic spontaneous urticaria
CyS	Systemic cyclosporine
ESR	Erythrocyte sedimentation rate
FcεRI	High-affinity IgE receptor
Gi	Gastrointestinal involvement
HCQ	Systemic hydroxychloroquine
HR	High response
HUV	Hypocomplementemic urticarial vasculitis
IgA	Immunoglobulin A
IgE	Immunoglobulin E
IgM	Immunoglobulin M
Jo	Joint involvement
LR	Low response
MR	Moderate response
MTX	Systemic methotrexate
NR	No response
NUV	Normocomplementemic urticarial vasculitis
R	Relapse
SCCS	Systemic corticosteroids
SLE	Systemic lupus erythematosus
Th	Thyroid involvement
TrC	Treatment continuation (the patient continues the treatment)
TrD	Treatment discontinuation (the patient discontinues the treatment)
UCT	Urticaria control test
UV	Urticarial Vasculitis
W	Worsening of disease
Wh	Wheals
