# Supplementary material for: Challenging Clinical Therapeutic Approach to Urticarial Vasculitis: A Case Series
Source: J Clin Med. 2025 Jun 27;14(13):4580. doi: 10.3390/jcm14134580 (PMC12249814; doi:10.3390/jcm14134580)
Supplement: Supplementary file 1 [file jcm-14-04580-s001.zip › jcm-3674107-supplementary.pdf]

**Patient Name:**
**Date of Birth:**

Date: ______________________________

URTICARIA/ANGIOEDEMA (Hives/Swelling) CONTROL TEST

Instructions: You have urticaria. The following questions should help us understand your current health situation. Please read through each question carefully and choose an answer that best fits your situation. Please limit yourself to the ***last four weeks***. *Please do not think about the questions for a long time,* and do remember to answer all questions and to provide *only one* answer to each question.

1.How much have you suffered from the **physical symptoms of the urticaria (itch, hives [welts], and or swelling)** in the last four weeks?					
☐ Very much 0	☐ Much 1	☐ Somewhat 2	☐ A little 3	☐ Not at all 4	
2. How much was your **quality of life** affected by the urticaria in the last four weeks?					
☐ Very much 0	☐ Much 1	☐ Somewhat 2	☐ A little 3	☐ Not at all 4	
3. How often was the **treatment** for your urticaria in the last four weeks **not enough** to control your urticaria symptoms?					
☐ Very often 0	☐ Often 1	☐ Sometimes 2	☐ Seldom 3	☐ Not at all 4	
4.**Overall,** how well have you had your urticaria **under control** in the last four weeks?					
☐ Not at all 0	☐ A little 1	☐ Somewhat 2	☐ Well 3	☐ Very well 4	
**TOTAL SCORE:**					

Reproduced from Weller K, Groffik A, Church MK, et al. Development and validation of the Urticaria Control Test: A patient reported outcome instrument for assessing chronic urticaria. J Allergy Clin Immunol 2014; 133:1365

A score of 16 indicates complete disease control. A score of <12 on the UCT identifies patients with poorly controlled chronic urticaria (CU), and a score of ≥12 identifies those with well-controlled symptoms. An improvement in 3 points is a minimal response, and an improvement of ≥6 points is a marked response.
